# Measurement Reduction Methods for Processing Tomographic Images

**DOI:** 10.3390/s23020563

**Published:** 2023-01-04

**Authors:** Alexey I. Chulichkov, Dmitriy A. Balakin

**Affiliations:** Faculty of Physics, M. V. Lomonosov Moscow State University, Leninskie Gory, 1, Bld 2, Moscow 119991, Russia

**Keywords:** tomography, image processing, measurement reduction, optimal estimation, 07.05.Kf

## Abstract

The importance of development of new methods for reconstruction of an object image given its sinogram and some additional information about the object stems from the possibility of artifact presence in the reconstructed image, or its insufficient sharpness when the used additional information does not hold. The problem of recovering artifact-free images of the studied object from tomography data is considered in the framework of the theory of computer-aided measuring systems. Methods for solving it are developed. They are based on narrowing the class of possible images using less artifact-inducing information. An example of such information is the natural condition of non-negativeness of the estimated brightnesses. The main problem that arises is the large dimensionality of the images, which prevents the use of direct algorithms. One proposed method is based on local approach, namely correction of the result of unfiltered backprojection by applying a locally (in the space of the output image) optimal linear transformation. Another method processes a sinogram directly, without using backprojection, using iterative implementation of the measurement reduction technique. Examples of use of the proposed methods for processing teeth sinograms are given.

## 1. Introduction

Despite the widespread use of computed tomography (CT) to recover the internal structure of a sample, the problem of improving the recovery quality given the data of its tomography measurements (sinogram) remains an active research field [1,2,3,4,5,6,7,8,9]. The essence of tomography is the relation between the spatial structure of the object (in the case of X-ray tomography, this is the spatial distribution of the absorption coefficient of X-rays) and its sinogram formed using the projections of that structure. Its mathematical model is usually the Radon transform operator. Direct inversion of this operator is not always possible due to the problem being underdetermined. The reason for this is that the number of sensors multiplied by the number of rotations of the sensor array is usually less than the number of pixels of the reconstructed image of the desired resolution. Furthermore, the matrix of the resulting operator is often quite large.

Existing methods of solving this problem usually fall into two groups [10,11,12]. The first group consists of methods that take advantage of the Fourier slice theorem to calculate the inverse of Radon transform ([13], Ch. 3). In the continuous case this allows exact inversion, but in the discrete case distortions arise, so additional filtering is made to improve image quality and suppress the influence of measurement errors. The second group consists of algebraic reconstruction techniques ([13], Ch. 7; in particular, SART [14]). They treat the problem as the problem of solving a large sparse system of linear equations. Usually, iterative methods are used for this purpose. In addition, regularization is also often employed to make the solution more stable. Regularization is equivalent to use of additional information about the object such as the smoothness of the spatial distribution of its characteristics. If that information is not accurate for the processed object, its reconstructed image might contain artifacts or be insufficiently sharp. As a result, images with artifacts, or with insufficient resolution, etc. are obtained.

This article attempts to apply the methods of the theory of computer-aided measuring systems [15,16] to the problem of recovering an image of the studied object given its sinogram. The aim is to take advantage of the structure of Radon transform operator to improve recovered image quality under the following constraints. On one hand, using information about the object that may be difficult to verify is to be avoided. An example of such information is smoothness of the spatial distribution of the characteristic of interest. On the other hand, the non-negativity of absorption coefficients or, more generally, the fact that the spatial distribution of absorption coefficients belongs to a given set (like the nonnegative cone), similar to [17,18], is to be taken into account. In addition, the aim is recovery of an object image, not extraction of specific features of an image of the studied object as in for example, [19,20,21]. We also note that the proposed method is purely computational and does not require a specific tomography setup like in optical coherence tomography [19,20].

## 2. Materials and Methods

### 2.1. Linear Model of Sinogram Registration

Let us build a mathematical model of recording a sinogram that takes into account the finite number of projections and the finite size of the sensors that register the radiation that has passed through the sample. Consider a tomography setup in which radiation is detected by an array of *J* sensors that have finite linear size *d*. A parallel beam of rays is incident on the sample. The radiation attenuated by the sample is recorded by a sensor. Attenuation is modeled by integration of the distribution of sample absorption coefficient f(·,·) along the path of rays entering the sensor. The sample is rotated in steps, usually with uniform angular step size Δφ, and measurements are taken after each repositioning of the sensors until the angular position of the sample w. r. t. the sensor array changes by at least $180^{\circ }$. The resulting dataset is called a sinogram.

Let the sample absorption coefficient distribution f(x,y) be equal to zero outside a certain region Ω that entirely lies within the radiation ray paths for all rotation angles. Denoting by *j* the sensor number and by *k* the rotation angle number, we obtain that the output signal $g_{k,j}$ of the *j*th sensor at the *k*th rotation angle is the integral of the absorption coefficient f(x,y) over a strip of width *d* located in front of the *j*th sensor, rotated by an angle $\varphi_{k}=\left(k-1\right)\Delta \varphi$, j=1,⋯,J, k=1,⋯,K:

(1) $$g_{k,j}=\int_{D_{k,j}}f\left(x,y\right)dxdy=\int_{\Omega }f\left(x,y\right)a_{k,j}\left(x,y\right)dxdy=\left(a_{k,j},f\right).$$

Here $D_{k,j}$ is a strip with width *d* rotated by the angle $\varphi_{k}$, and $a_{k,j}\left(x,y\right)$ is the indicator function of this region. Now the result of registering a sinogram can be written as

(2) $$\xi_{k,j}=g_{k,j}+\nu_{k,j}={\left(Af\right)}_{k,j}+\nu_{k,j},$$

where $\nu_{k,j}$ are measurement errors. The linear operator *A* in (2) maps the function $f\in L^{2}\left(\Omega \right)$ to a vector with coordinates $g_{k,j}$, j=1,⋯,J, k=1,⋯,K.

Before proceeding to processing methods, we note that parallel projection is not necessary for the application of the following methods. One of the reasons we consider the parallel projection is because the experimental data analyzed in Section 3 were obtained for that geometry. The main reason, however, is the relative simplicity of this case. For fan projections (cone-beam geometry), both for equiangular and equispaced rays weights have to be introduced in the backprojection method below (see, e.g., [13], Sections 3.4 and 3.5). Furthermore, an algorithm for rearranging fan beam projection data into equivalent parallel beam projection data (naturally, not uniformly sampled) exists (ref. [13], Section 3.4.3).

### 2.2. Backprojection Method

A common simple method for reconstructing the spatial distribution of the absorption coefficient of the sample is the backprojection method. Its estimate is given by

(3) $$\hat{f}\left(x,y\right)=\sum_{k,j}a_{k,j}\left(x,y\right)\xi_{k,j}$$

and it does not use any additional information.

**Figure 1:**
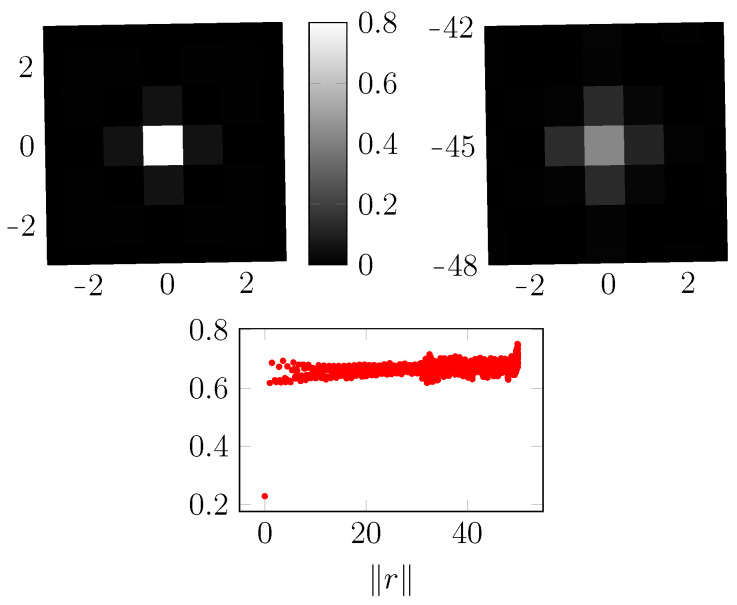
**Upper** part: magnified (5×5 pixel) nonzero parts of filtered backprojection of a 100×100 pixel sinogram (100 uniformly distributed projection angles, 100 sensors) of a point object located in the center (**upper left**) or near the border (**upper right**) of the field of view of the sensor array. **Lower** part: the norm of the difference of filtered backprojection output for a sinogram of a point object as a function of the distance from the position of the object to the center. As both coordinates change from −50 to 50, the distance ∥r∥ from the center to the point changes from 0 to 50. The plot shows that the blurring of the filtered backprojection result of a sinogram of a point object does not only depend on the distance from the object (if it did, there would be a single, not necessarily horizontal, line, instead of two lines). In particular, the single red dot at ∥r∥=0 corresponds to much lower blurring of the filtered backprojection result of a sinogram of a point object at the center, corresponding to the upper left image.

We note that in the continuous case, the filtered version of backprojection with the ramp filter (the factor in the integrand of the integral replacing the sum in (3) in the continuous case that is equal to the absolute value of the position in the sinogram) provides an algorithm that accurately recovers the object image given the sinogram measured for all angles and for all possible sensor positions in the array in the absence of noise (ref. [13], Section 3.3). However, using the ramp filter in the discrete case can cause artifacts for an insufficient number of rotation angles (see, e.g., Figure 3.10 in [13]) and the resulting reconstruction method is sensitive to noise. The use of different filters can reduce the influence of noise, but may introduce blurring or artifacts such as ringing near sharp changes in the absorption coefficient. Furthermore, the result of Radon transform, modeling the formation of a sinogram, followed by filtered backprojection slightly differs from the image of the object, causing blurring; see Figure 1. The blurring is different at different points of the field of view of the sensor array. It mostly depends on the distance to its center but it is not fully determined by that distance. This makes the result of filtered backprojection unsuitable for the local method described below in Section 2.4.1 as the initial estimate. If one has to solve the problem (11) or (12) for many positions in the field of view, most of the computational advantages over the direct approach vanish.

### 2.3. Elements of the Theory of Computer-Aided Measuring Systems

In the typical measurement setup considered in the theory of computer-aided measuring systems [15,16], the spatial distribution f∈F of the features of the object (for example, its absorption coefficient distribution) is transformed into the measurement result ξ∈X (in this case, a sinogram) by the measuring transducer according to the relation

(4) ξ=Af+ν,

which corresponds to (2) in the vector notation. In (4), Af is the proper result of the transformation, the linear operator A:F→X models the transducer, and ν is the random measurement error vector with known expectation Eν=0 and known covariance operator $\Sigma_{\nu }:\forall z\in X\;\Sigma_{\nu }z=E\nu \left(z,\nu \right)$.

An attempt to determine *f* as the solution of the equation ξ=Af leads to an ill-posed problem [22], the main feature of which is the instability of the solution w. r. t. errors in acquired image ξ. One of the widely used approaches to overcome this difficulty is the regularization of the problem, as a result of which the solution of the regularized problem continuously depends on ν. The solution is obtained by minimizing the sum of the residual functional ${∥\xi -Af∥}^{2}$ and the stabilizer. Due to the practical importance of such methods, their development remains relevant [2,23,24,25,26]. However, the regularized least squares functional is not, in general, related to estimation error.

In contrast to this approach, in the theory of computer-aided measuring systems one looks for the transformation (in a given class of transformations) of the input data whose result can be interpreted as the most accurate estimate of the feature of interest of the research object. In addition, the theory of computer-aided measuring systems allows one to quantitatively characterize [15,16] the degree of consistency of the mathematical model of the measurement scheme (4), the measurement result and the produced estimate. To specify the relation between the feature of interest and the properties of the object represented by the vector *f* in (4), the researcher formulates the model of an ideal measuring transducer. Its input signal *f* is the same as that of the real measuring transducer, but its output signal Uf is equal to the features of interest of the research object. In the considered case, Uf can be the pixelized version of the absorption coefficient distribution.

For simplicity, let the spaces X, F and U be finite-dimensional and Euclidean. The values of coordinates of ξ are brightnesses of corresponding pixels of the processed sinogram. Then the linear operator $R_{*}:X\to U$ implementing the linear measurement reduction method is defined as the one minimizing the worst-case (in *f*) mean-squared error (MSE) of using Rξ as the estimate of Uf:

$$h\left(R\right)=\underset{f\in F}{\sup}E{∥R\xi -Uf∥}^{2}∼\min_{R:X\to U}.$$

As shown in [15], the MSE is minimal for

(5) $$R_{*}=U{\left(\Sigma_{\nu }^{-1/2}A\right)}^{-}\Sigma_{\nu }^{-1/2}$$

if the condition

(6) $$U\left(I-A^{-}A\right)=0$$

is satisfied, in which case the minimal MSE is

(7) $$h\left(R_{*}\right)=\mathrm{tr}U{\left(A^{*}\Sigma_{\nu }^{-1}A\right)}^{-}U^{*}.$$

Here ${}^{-}$ denotes pseudoinversion (Moore-Penrose inversion).

If the condition (6) is not satisfied, the value of $R_{*}\xi$ is, nevertheless, the optimal (in the above sense) estimate of $UA^{-}Af$ with no further information possible to extract from the measurement ξ. In other words, two objects $f_{1}$ and $f_{2}$ whose characteristics $Uf_{1}$ and $Uf_{2}$ differ only by a vector *q* satisfying $U\left(I-A^{-}A\right)q=0$ cannot be distinguished on the basis of their measurements alone.

In the text below, we consider U=I, so the object is considered to be already pixelized, with constant values of the feature of interest (e.g., the absorption coefficient) throughout each pixel, and the objective is to recover the pixelized distribution. As a result, the operator modeling the ideal measuring transducer is the identity operator, U=I. Otherwise, we would have to consider $F=L^{2}\left(\Omega \right)$ and include the discretization into the operator *U*. Please note that, generally, the discretization does not have to be done, e.g., by selecting the values at the nodes of a rectangular grid.

A variation of the above approach can be obtained by considering that for an arbitrary linear transformation *R*

Rξ=RAf+Rν=Uf+(RA−U)f+Rν,

so Rξ is interpreted as ideal image Uf that is distorted by random noise Rν and the “false signal” (RA−U)f depending on the unknown vector *f*. Let us consider the effect of the noise to be negligible if the mean-squared norm of the noise term is below a given threshold ε, $E{∥R\nu ∥}^{2}={∥R\Sigma_{\nu }^{1/2}∥}_{2}^{2}\leq \epsilon$, where ${∥\cdot ∥}_{2}$ is Hilbert—Schmidt operator norm. The smaller the term (RA−U)f, the smaller the value ${∥RA-U∥}_{2}^{2}$ for any value of the unknown *f*. Thus, it is reasonable to choose *R* as a solution to the minimization problem

(8) $$\underset{R:X\to U}{\inf}\left\{{∥RA-U∥}_{2}^{2}|{∥R\Sigma_{\nu }^{1/2}∥}_{2}^{2}\leq \varepsilon \right\}.$$

Its solution is given in the monograph [15] and it is

(9) $$R_{\varepsilon }=\left\{\begin{array}{cc} UA^{*}{\left(AA^{*}+\omega \Sigma_{\nu }^{2}\right)}^{-1} & 0<\varepsilon <\sigma^{2}\left|\right|A^{-}{\left|\right|}_{2}^{2}\\ R_{*}, & \epsilon \geq h(R_{*}), \end{array}\right.$$

where the parameter ω>0 is chosen so that the equality ${∥R\Sigma_{\nu }^{1/2}∥}_{2}^{2}=\varepsilon$ holds. ω is the Lagrange parameter in the conditional optimization problem (8), so it can also be chosen directly from a compromise between lowering ${∥RA-U∥}_{2}^{2}$ and lowering ${∥R\Sigma_{\nu }^{1/2}∥}_{2}^{2}$.

### 2.4. Estimate Improvement Using Pixel Non-Negativity

One can reduce the estimation error of image *f* by narrowing the class of possible “ideal” images of the scene. For example, one can take into account that the brightness of each pixel of the image is non-negative. Therefore, the vector *f* belongs to the non-negative cone $F_{+}$: $f\in F_{+}={\left[0,\infty \right)}^{\dim F}\subset F$ (or, more generally, *f* belongs to a convex closed set). The refined estimate is defined [17,18] as the fixed point of the following transformation of an estimate $\hat{u}$.

- The linear reduction estimate $R_{*}\xi$ and an estimate $\hat{u}$ are combined by considering $\left(\begin{array}{c} \xi \\ \hat{u} \end{array}\right)$ as a measurement (composed of the real measurement and a dummy measurement) made by a MT $\left(\begin{array}{c} A\\ U \end{array}\right)$ and affected by noise with covariance operator $\left(\begin{array}{cc} \Sigma_{\nu } & 0\\ 0 & \Sigma_{R_{*}\xi } \end{array}\right)$, where $\Sigma_{R_{*}\nu }=R_{*}\Sigma_{\nu }R_{*}^{*}$.
- The previous result is projected onto $F_{+}$.

For calculation, the initial value of the estimate is the linear reduction estimate projected onto $F_{+}$. The method in [17] used ordinary projection by minimizing Euclidean distance $∥\cdot ∥$, while minimization of Mahalanobis distance $∥\Sigma_{R_{*}\xi }^{-1/2}\cdot ∥$ associated with the covariance operator of the reduction estimate $\Sigma_{R_{*}\xi }^{}=U{\left(A^{*}\Sigma_{\nu }^{-1}A\right)}^{-}U^{*}$ in [18] showed faster convergence at the cost of more complex projection.

**Figure 2:**
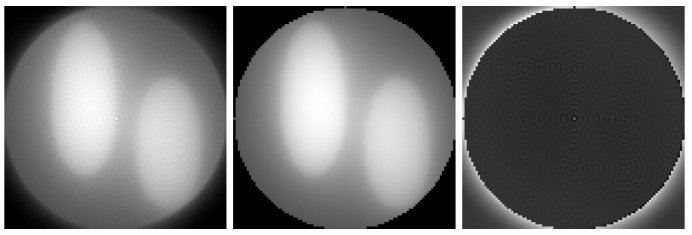
**Left**: the adjoint operator $A^{*}$ applied to a sinogram of a phantom structure (see Figure 9a for the sinogram and the left part of Figure 3 for the structure itself). **Center**: the result of unfiltered backprojection of the same sinogram. **Right**: the difference of preceding images after multiplying the result of unfiltered backprojection by a constant factor to minimize the difference. In the continuous case, the constant factor is 2π and the difference is zero. In the discrete case, one can see the differences outside the reconstruction circle Ω and near the center of the field of view.

Three difficulties arise when implementing the above version of the measurement reduction method directly. First, the matrix of the operator *A* is large when the sinogram size is large, making direct methods of calculating $R_{*}\xi$ unsuitable due to long calculation time. Second, while the product Ag of *A* and an arbitrary vector g∈F (Radon transform of the vector) is relatively easy to calculate, it is not so for the adjoint operator $A^{*}$ (it is close, but not identical to the scaled operator implementing unfiltered backprojection, see Figure 2). Third, the matrix of the covariance operator $\Sigma_{R_{*}\xi }$ of the linear reduction estimate is usually quite large. It is larger than the matrix of the operator *A* if the problem is underdetermined, as it usually is in the case of tomography with a few angular positions of the sensors. Finally, it is also less sparse than the matrix of the operator *A*. For a fixed angle of the sensor array, a single pixel of the object usually affects the output of a small portion of sensors. Furthermore, non-negativity is specific to the natural basis. For that reason, the projection-based approach above prevents taking advantage of possible symmetries of the measuring scheme (e.g., translational symmetry) to simplify the calculations (e.g., using fast Fourier transform).

In the following sections, let us suppose, for simplicity, that the noise affecting measurement data is white, $\Sigma_{\nu }=\sigma^{2}I$. If this is not true, but the noise covariance operator $\Sigma_{\nu }$ is non-degenerate, one can replace ξ with $\Sigma_{\nu }^{-1/2}\xi$ and *A* with $\Sigma_{\nu }^{-1/2}A$. As the matrix of the noise covariance operator is often sparse or even diagonal, this usually can be done efficiently. In addition, $a_{k,j}^{*}$ is the row of *A* as an 1D array, i.e., the contributions of various pixels of the studied object to the output of *j*th sensor at *k*th projection angle.

#### 2.4.1. Local Approach

One way of dealing with the above difficulties is based on the following idea [8,27]. Let the distortion introduced by a linear image registration system be such that the image of a point object is a spot. Then the information about the brightness of a given pixel of object *f* is mostly contained in a fragment of the image η covering this spot. For linear estimation of the brightness of this pixel of an ideal image Uf, it is sufficient to use a linear combination of the values of image pixels $\eta_{i,j}$ of the image η that are near the position corresponding to the object pixel to be recovered. The weights $r_{i,j}$ of the linear combination are selected by minimizing the estimation error. Let us suppose that the spot for a point object located at the origin, with $f_{i,j}=\delta_{i,j}=\left\{\begin{array}{cc} 1, & i=j=0,\\ 0, & \mathrm{otherwise}, \end{array}\right.$ fits into the (2M+1)×(2M+1) pixel rectangular window centered at the origin. In other words, for the point object above, without noise one would have $\eta_{i,j}=0$ if |i|>M or |j|>M. Let us also suppose that the imaging system has translational invariance, that is, the image of an object *f* is produced according to

(10) $$\eta_{ij}=\sum_{i^{\prime },j^{\prime }=-M}^{M}b_{i^{\prime },j^{\prime }}f_{i-i^{\prime },j-j^{\prime }}+\mu_{ij},$$

where μ is the noise of the image registration system. Since Equation (10) is similar to (4), we can use the methods of the theory of computer-aided measuring systems to produce an estimate of the ideal image of the research object. In other words, the operator *B* in (10) corresponds to the linear transformation of the image of a point object located at the origin into the result of unfiltered backprojection of its sinogram. The measurement of the sinogram is simulated using the same number of sensors and the same projection angles as for the real sinogram.

For an estimate of the brightness of the object image at the point (0,0), we use the brightnesses of the pixels with indices {−M≤i≤M, −M≤j≤M}. We use a linear estimate of $f_{0,0}$ that is of the form

$${\hat{f}}_{0,0}=\sum_{i=-M}^{M}\sum_{j=-M}^{M}r_{i,j}\eta_{i,j}.$$

We find the coefficients $r_{i,j}$, −M≤i≤M, −M≤j≤M, by requiring that the convolution of the blurry image $b_{k,l}$ of a point object with a mask $r_{k,l}$, k,l=−M,⋯,M, should give an image of a point object:

(11) $$\sum_{i=-M}^{M}\sum_{j=-M}^{M}r_{i,j}b_{i-n,j-m}=\delta_{0,n}\delta_{0,m},m,n=-M,\cdots ,M.$$

If these linear equations w. r. t. $r_{k,l}$, k,l=−M,⋯,M, expressing the condition of unbiasedness of the brightness estimate $f_{0,0}$, have a unique solution, then the coefficients $r_{k,l}$, k,l=−M,⋯,M, are uniquely determined. If the system (11) is degenerate or if the estimation error is unacceptably large, one should do the same as in the formulation and solution of problem (8), i.e., minimize

(12) $$\sum_{n,m}^{}{\left(\sum_{i=-M}^{M}\sum_{j=-M}^{M}r_{i,j}b_{i-n,j-m}-\delta_{0,n}\delta_{0,m}\right)}^{2}$$

with constraint on the estimation error $\sum_{i=-M}^{M}\sum_{j=-M}^{M}\sigma^{2}r_{i,j}^{2}\leq \varepsilon$ (as mentioned in the previous subsection, we consider that $\Sigma_{\nu }=\sigma^{2}I$).

An estimate of the brightness of each pixel of the image *f* is obtained by successive application of the proposed algorithm to each pixel of the image *f*, placing it at the origin:

(13) $${\hat{f}}_{i,j}=\sum_{i^{\prime },j^{\prime }=-M}^{M}r_{i^{\prime },j^{\prime }}\eta_{i-i^{\prime },j-j^{\prime }}.$$

An image produced by unfiltered backprojection approximately satisfies the above conditions, so it can be used as the image η. In this case, the operator *B* is the product of an operator *A* in (4) that transforms a spatial distribution of absorption coefficient into a sinogram and an operator transforming a sinogram into the result of its unfiltered backprojection.

To take non-negativity into account, the following additional operations are performed. Suppose that, using this method, we estimate the brightness of the image $\hat{f}$, and for some pixel (denote its index as (0,0)) its value ${\hat{f}}_{0,0}<0$. Since pixel brightness values a priori cannot be negative, it can be considered that an exact “additional measurement” of pixel brightness $f_{0,0}$ was carried out, the result of which is written as ${\hat{f}}_{0,0}=0$.

The values of the pixels of the estimate $\hat{f}$ are correlated, so this additional measurement should also affect the estimated brightnesses of the surrounding pixels $f_{k,l}$. As shown in, e.g., [15], if the expectations, variances, and covariance of two random variables, ${\hat{f}}_{k,l}$ and ${\hat{f}}_{0,0}$, are known, then the best mean-squared linear estimate of the random variable ${\hat{f}}_{k,l}$ from the observation of the random variable ${\hat{f}}_{0,0}=0$ is ${\hat{\hat{f}}}_{k,l}=E{\hat{f}}_{k,l}+\frac{\mathrm{cov}({\hat{f}}_{k,l},{\hat{f}}_{0,0})}{D{\hat{f}}_{0,0}}\left(0-M{\hat{f}}_{0,0}\right)$, and the variance of this estimate is $D{\hat{\hat{f}}}_{k,l}=D{\hat{f}}_{k,l}-\frac{\mathrm{cov}{\left({\hat{f}}_{k,l},{\hat{f}}_{0.0}\right)}^{2}}{D{\hat{f}}_{0.0}}$. According to the measurement model (4), (10) and its reduction (5), (13) to the ideal image, the covariance $\mathrm{cov}({\hat{f}}_{k,l},{\hat{f}}_{0,0})$ and variance $D{\hat{f}}_{0,0}$ are $\mathrm{cov}\left({\hat{f}}_{k,l},{\hat{f}}_{0,0}\right)=\sigma^{2}\sum_{i=-M}^{M}\sum_{j=-M}^{M}r_{i-k,j-l}r_{i,j}$ and $D{\hat{f}}_{0,0}=\sigma^{2}\sum_{i=-M}^{M}\sum_{j=-M}^{M}r_{i,j}^{2}$, respectively. Replacing the unknown expectations $M{\hat{f}}_{k,l}$ and $M{\hat{f}}_{0,0}$ by their estimates ${\hat{f}}_{k,l}$ and ${\hat{f}}_{0,0}$, we obtain a refined estimate of the pixels ${\hat{f}}_{k,l}$ of the image *f* in the vicinity of (0,0), k,l=−M,⋯,M:

$${\hat{\hat{f}}}_{k,l}={\hat{f}}_{k,l}+\frac{\sum_{i=-M}^{M}\sum_{j=-M}^{M}r_{i-k,j-l}r_{i,j}}{\sum_{i=-M}^{M}\sum_{j=-M}^{M}r_{i,j}^{2}}\left(0-{\hat{f}}_{0,0}\right).$$

This procedure is applied each time a component of the estimate is found to be negative.

The resulting Algorithm 1 is shown below.
**Algorithm 1:** Local algorithm for measurement reduction of a sinogram
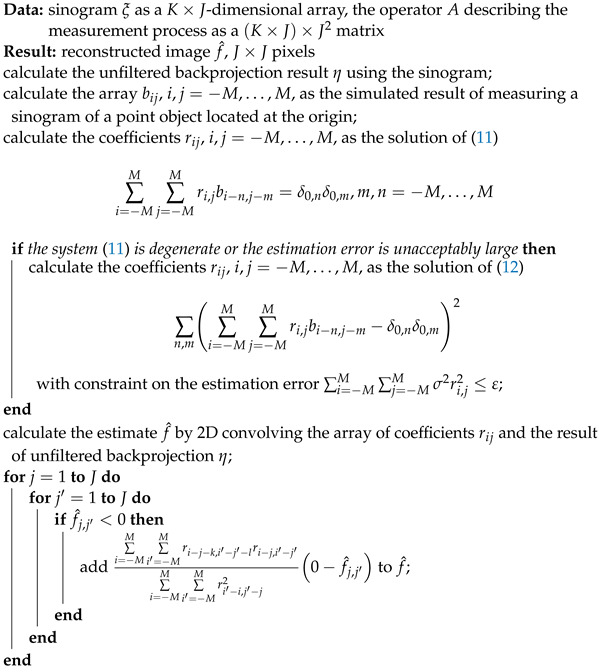


The choice of *M* in the above method presents a tradeoff between improving clarity and reducing computation time. Therefore, in principle, in the above approach one should use as large *M* as possible within the constraints on processing time and available memory size. Moreover, in the presence of noise the contrast is usually reduced when increasing *M*, as it also results in more extreme values of the noise in the produced image.

As mentioned above, in the case of reconstruction from fan projections, the backprojection step has to be replaced by weighted backprojection. The remainder of the algorithm only uses the backprojection results, so there are no other changes. This remainder can be considered as a correction of the backprojection result, similar to, e.g., the compensation done by the neural network in [28].

#### 2.4.2. Iterative Approach

The difficulties of the direct approach can also be solved using iterative, and preferably matrix-free, calculation methods. Iterative methods enable an additional improvement in calculation time. Instead of (iteratively) calculating and then projecting the reduction result onto the non-negative cone $F_{+}$, one can do the projection at each iterative step. This also reduces the downside of projection by minimizing Euclidean distance instead of Mahalanobis distance.

An example of an iterative method that is suitable for solving this problem is Kaczmarz’s method, see, e.g., (Section 12.4 of [29]). The Algorithm 2 for reduction using Kaczmarz’s method is as follows [30].
**Algorithm 2:** Iterative algorithm for measurement reduction of a sinogram, basic version
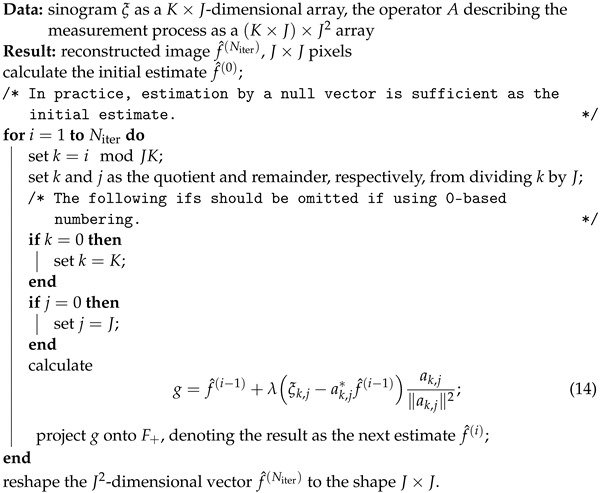


λ is a positive scalar between 0 and 2 that can be considered as the step size of the algorithm. High values of λ improve convergence, but can result in numerical instability.

On one hand, this algorithm is similar to the recurrent version of measurement reduction (see [15], Section 5.5), in which the linear update (cf. (14)) takes the form

$$g={\hat{f}}^{\left(i-1\right)}+\left(\xi_{i}-a_{i}^{*}{\hat{f}}^{\left(i-1\right)}\right)\frac{{\left(A^{\left(i-1\right)*}A^{\left(i-1\right)}\right)}^{-1}a_{i}}{1+a_{i}^{*}{\left(A^{\left(i-1\right)*}A^{\left(i-1\right)}\right)}^{-1}a_{i}},$$

$${\left(A^{\left(i\right)*}A^{\left(i\right)}\right)}^{-1}={\left(A^{\left(i-1\right)*}A^{\left(i-1\right)}\right)}^{-1}-\frac{{\left(A^{\left(i-1\right)*}A^{\left(i-1\right)}\right)}^{-1}a_{i}a_{i}^{*}{\left(A^{\left(i-1\right)*}A^{\left(i-1\right)}\right)}^{-1}}{1+a_{i}^{*}{\left(A^{\left(i-1\right)*}A^{\left(i-1\right)}\right)}^{-1}a_{i}}.$$

In these equations, *A* is assumed to have the shape $KJ\times J^{2}$ with arbitrary but fixed raveling of multi-indices (k,j). $A^{\left(i\right)}$ is the part of *A* with rows up to and including the *i*th, ${\left(A^{\left(i\right)*}A^{\left(j\right)}\right)}^{-1}$ is the scaled covariance operator of the estimate in the step *i*, and the iterations stop after accounting for each measurement once. Aside from fixed number of iterations, as one can see, the main difference with calculations using Kaczmarz’s method is the use of the estimate covariance operator. However, the above linear update is valid only if the covariance operator of the initial estimate is already non-degenerate. This is not the case during at least first iterations. Furthermore, the formulas for the update step in the general case are cumbersome. Nevertheless, the main drawback of using the recurrent version of measurement reduction for this purpose is the requirement of keeping the covariance operator of the estimate in memory.

On the other hand, the proposed reduction algorithm is also quite close to the algebraic reconstruction technique (ART), which also uses Kaczmarz’s method to invert Radon transform. Unlike ART, the proposed algorithm does not use filtering or regularization. Nevertheless, the similarity allows borrowing some techniques used in ART-like algorithms to improve numerical stability. One of those is reordering the angles at which measurements were taken according to the golden ratio method [31], making consequent measurements less correlated. Another is simultaneous update of the estimate by the total corrections calculated for all rays in the projection and for the same estimate. The reason for this is that different rays with the same rotation angle do not interact and the cross-influence is a side effect of the used interpolation method. A similar difference exists between simultaneous algebraic reconstruction (SART) and ART. Hence, the revised version of the proposed algorithm is Algorithm 3. Instead of a single loop over individual measurement components, there is now an outer loop over reordered sensor array rotations and an inner loop over the sensors in the array, where an update of the estimate is calculated.
**Algorithm 3:** Iterative algorithm for measurement reduction of a sinogram, improved version
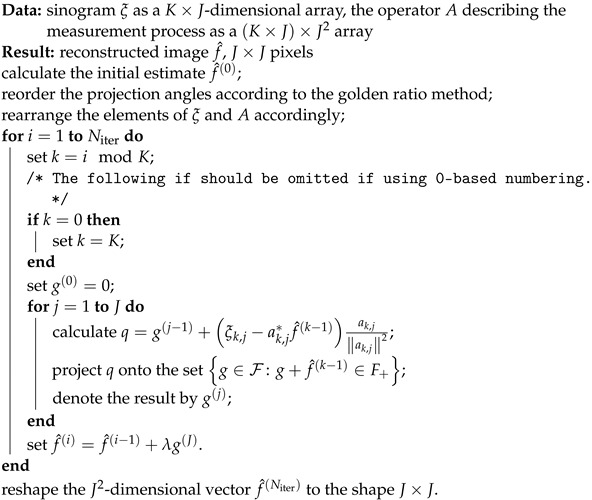


For comparison, in SART when upper and/or lower bounds on pixel values are introduced, projection takes place in the outer loop.

One can also use a different iterative method, for example, gradient descent [29] (also known, for this specific purpose, as the Landweber method [32]). The main drawback is the need for the adjoint operator $A^{*}$. While its full matrix is not required and a method for reasonably fast and memory-efficient way of calculating its product with an arbitrary vector is sufficient, as the update step (replacing (14)) is given by

$$g=f^{\left(i-1\right)}-\lambda A^{*}\left(Af^{\left(i\right)}-\xi \right)$$

for some values of the step size parameter $0<\lambda <2/{∥A∥}^{2}$, the use of unfiltered backprojection for that purpose causes residual blurring, as can be seen in Section 3. Furthermore, while the bounds on step size parameter of the Kaczmarz’s method are independent of the operator *A*, the upper bound on the step size parameter in the Landweber method depends on the largest singular value of *A*.

The projection in the proposed algorithms can be easily replaced by a more complex projection routine corresponding to richer information about the object. For example, one can have prior knowledge that the object is composed of a limited number of materials whose absorption coefficients do not vary. In other words, there are no smooth transitions between materials with different properties, similar to the discrete algebraic reconstruction technique [7]. In that case, one can augment the projection by also projecting on the set of mosaic images with the specified number of brightness levels, e.g., using the algorithm proposed in ([33], Ch. 5).

In the case of reconstruction from fan projections, as mentioned above, the necessary changes amount to rearranging the measurement results and the corresponding elements of the matrix *A*.

To conclude the section, Table 1 outlines the number of operations used in the proposed methods and the amount of used memory (in addition to the memory required to store the input data and the produced image, which is the same for all methods).

## 3. Results

Initial analysis of effectiveness of the proposed algorithms was carried out for simulated sinograms (100 angles, 100 sensors) of an object from [34].

**Figure 3:**
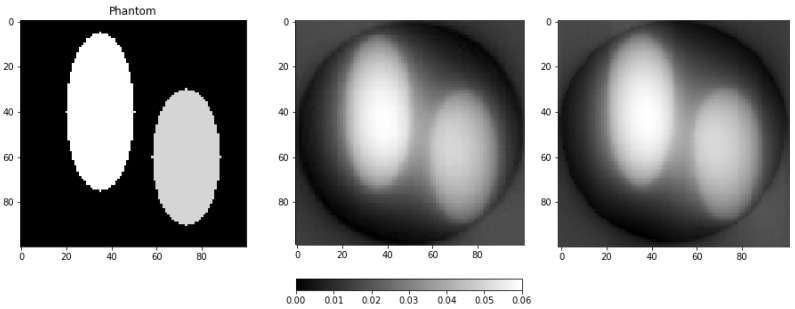
**Left**: a model image of the phantom structure (the same as the one used to produce Figure 2). **Center**: an estimate of the structure by the unfiltered backprojection method. **Right**: the estimate (9).

Figure 3 shows the model image and two results of linear methods: unfiltered backprojection and (9). It can be seen that the resulting images are blurry.

**Figure 4:**
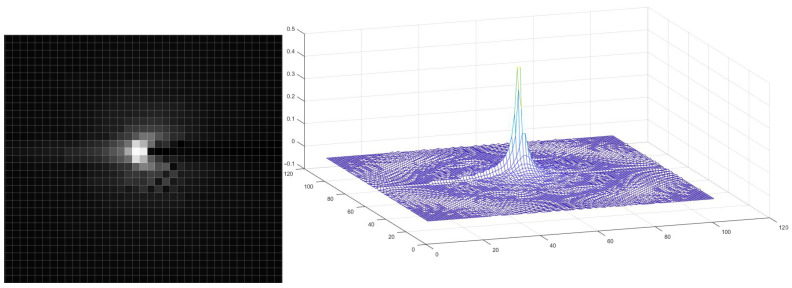
**Left**: the result of estimating the image of a point object placed at the origin using unfiltered backprojection. **Right**: a plot of the values of corresponding point spread function.

The matrix of the operator *B* from (10) was calculated by computer simulation of tomography of a point object placed at the origin and unfiltered backprojection of the simulated sinogram. The corresponding point spread function is shown in Figure 4. For simulation, the same number of sensors and the same projection angles as for the sinogram of the object in Figure 3 was used. The lack of isotropy is caused by discretization. For the specified number of sensors and projection angles, a good value of window size *M* is one for which most of the bright area fits into a (2M+1)×(2M+1) rectangle.

**Figure 5:**
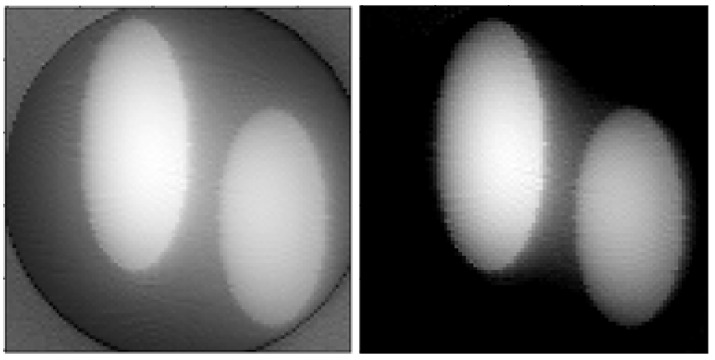
**Left**: a linear estimate of image *f* by the local method of increasing resolution. **Right**: refinement of the estimate taking into account the non-negative brightness of image *f*.

Linear estimation of the image *f* by the local approach method described in Section 2.4.1 for M=2 is shown on the left in Figure 5, and improvement of the estimate taking into account the non-negativity of its brightness is shown in Figure 5 on the right. It can be seen that the clarity of the image is higher when taking into account the non-negativity of its brightness than in Figure 3 and Figure 5 on the left.

**Figure 6:**
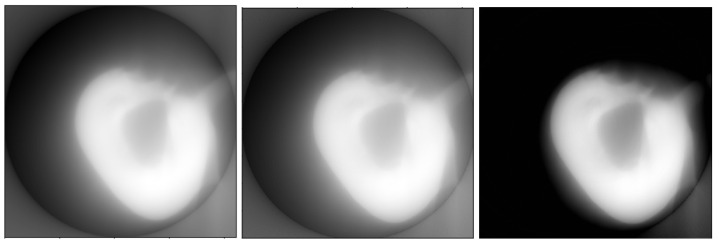
**Left**: tomography image of a tooth obtained using the unfiltered backprojection method. **Center**: a linear estimate using the local method of increasing the resolution. **Right**: improvement of the estimate taking into account the non-negativity of the image brightness for M=2.

**Figure 7:**
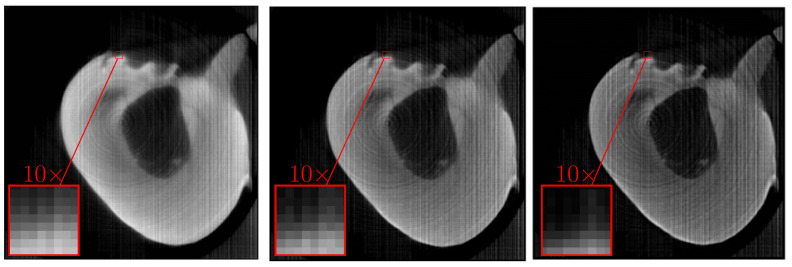
Distributions of the tooth absorption coefficient obtained by the local reduction method M=3 (**left**), M=5 (**center**) and M=7 (**right**). The negativity of the absorption coefficient is taken into account.

Similar results for the sinogram of a tooth (the sinogram was taken using a lost tooth which was not purposefully extracted) provided by the Laboratory for Reflectometry and Small-Angle Scattering of A. V. Shubnikov Institute of Crystallography, RAS, are shown in Figure 6 and Figure 7. A sinogram was obtained for 400 projection angles and 846 sensors. The produced images are 846×846 pixels. It can be seen that nonlinear refinement of the image estimate occurs in this case as well, leading to disappearance of artifacts and minor improvement of clarity.

Figure 7 shows the results of processing the same sinogram using different window size: with M=3 (left), M=5 (center) and M=7 (right). The estimate became sharper, but circular and linear artifacts appeared. However, possible reasons for their appearance are unequal detector sensitivities and inexact positioning of sensors. In other words, the assumed rotation angles and array positions can slightly differ from the actual ones. Meanwhile, slight remaining blurring masks the same effect when using previously shown methods. Resolution increases with increasing window size, but contrast decreases. Therefore, errors in the measurement model limit the upper value of *M* (in addition to the considerations mentioned at the end of Section 2.4.1) beyond which the quality of the produced image does not get better.

**Figure 8:**
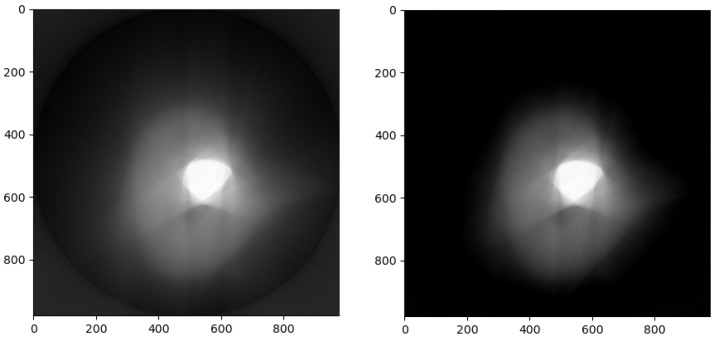
Distributions of the absorption coefficient of a tooth with a lead filling obtained by local reduction, M=2. **Left**: without taking into account the non-negativity of the absorption coefficient, **Right**: non-negativity is taken into account.

Figure 8 shows the results of reconstructing the absorption coefficient distribution using a sinogram of tooth with a lead filling (the sinogram was taken using a lost tooth which was not purposefully extracted), also provided by the Laboratory for Reflectometry and Small-Angle Scattering of the Institute of Crystallography named after A. V. Shubnikov, RAS (same data as presented in [35]), by the local reduction method with M=2. The sinogram is obtained for 400 angles by an array of 984 sensors. On the left is the estimate without taking into account the non-negativity of the absorption coefficients. On the right, the non-negativity is taken into account. Due to the wide range of changes in the absorption coefficient, an increase in sharpness when taking into account the non-negativeness is almost imperceptible. Please note that the artifacts in the form of a bright strip at the border of the filling, noted in [35], are not visible in the resulting image.

**Figure 9:**
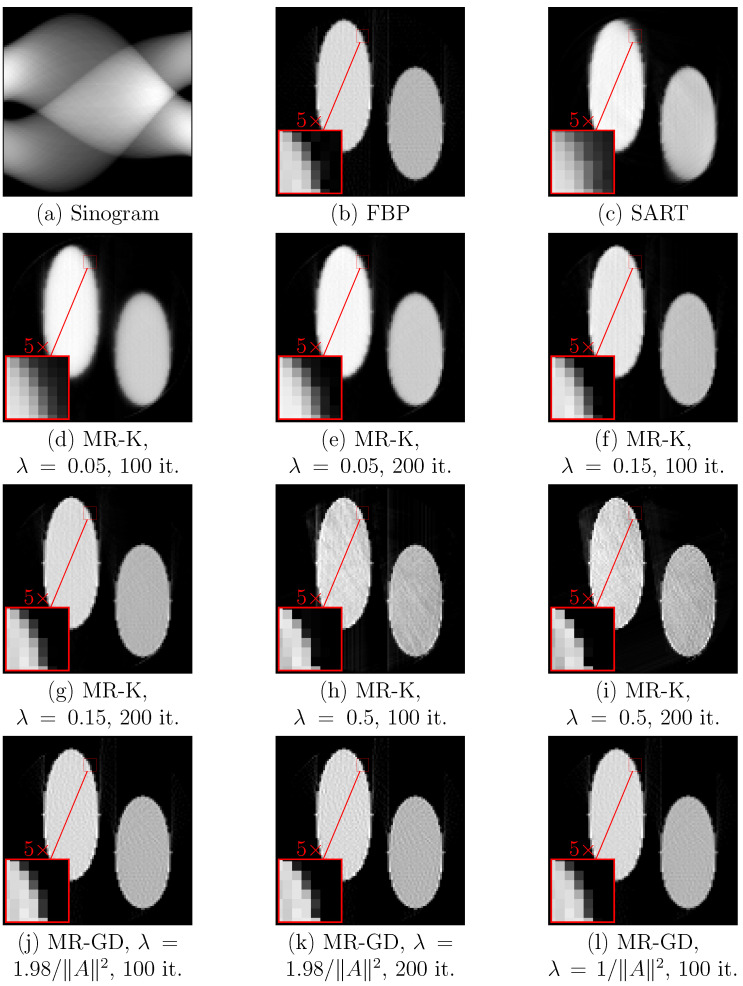
Processing results for (**a**) the sinogram (100×100 pixels) of a phantom structure using (**b**) filtered backprojection with ramp filter followed by clipping for non-negativity, (**c**) SART, (**d**–**i**) measurement reduction using Kaczmarz’s method with specified values of λ and numbers of iterations, (**j**–**l**) measurement reduction using gradient descent with specified values of λ and numbers of iterations. Zoomed-in insets show areas discussed in the text where the changes due to the choice of parameters are the most prominent.

**Figure 10:**
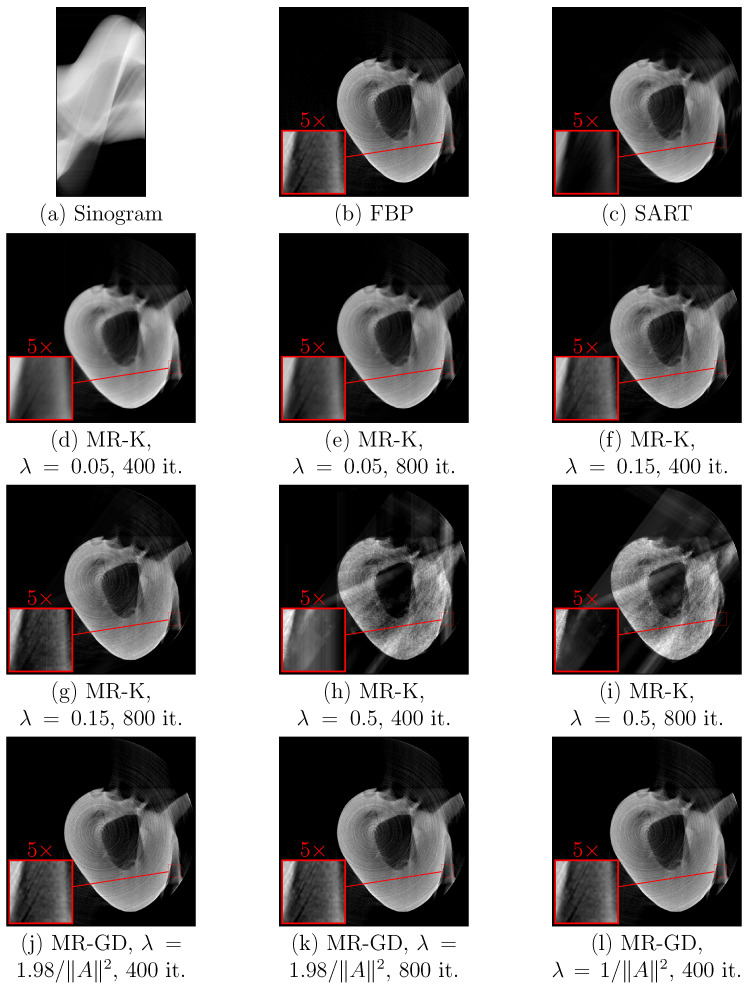
Processing results for (**a**) the sinogram (400×846 pixels) of a tooth using (**b**) filtered backprojection with ramp filter followed by clipping for non-negativity, (**c**) SART, (**d**–**i**) measurement reduction using Kaczmarz’s method with specified values of λ and numbers of iterations, (**j**–**l**) measurement reduction using gradient descent with specified values of λ and numbers of iterations. Zoomed-in insets show areas discussed in the text where the changes due to the choice of parameters are the most prominent.

**Figure 11:**
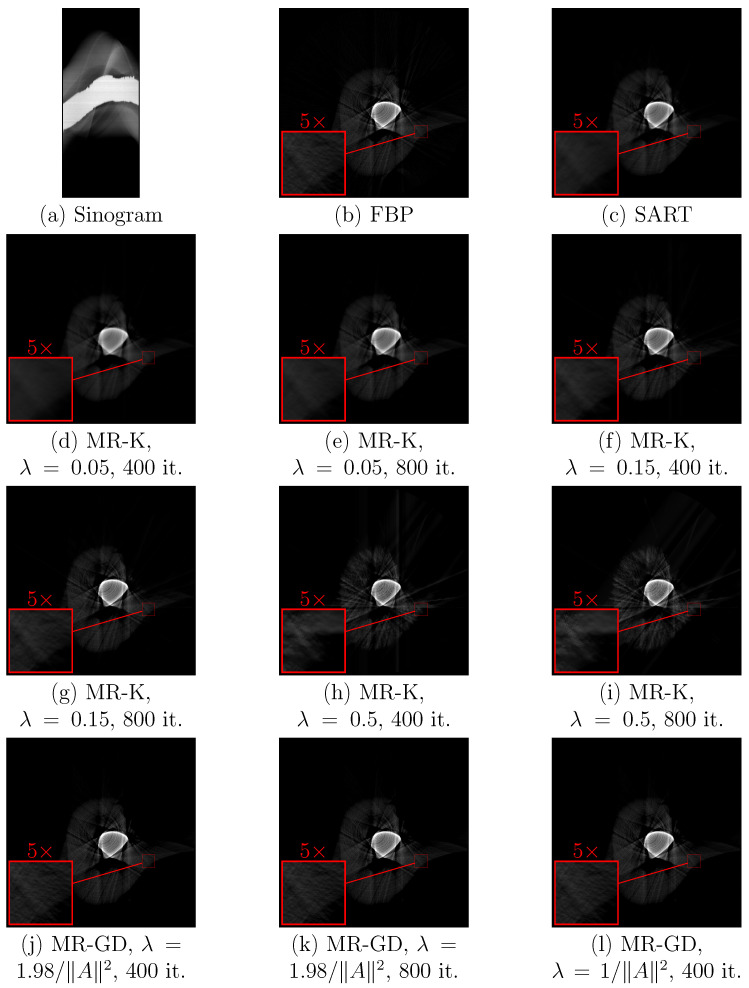
Processing results for (**a**) the sinogram (400 × 984 pixels) of a tooth with a lead filling using (**b**) filtered backprojection with ramp filter followed by clipping for non-negativity, (**c**) SART, (**d**–**i**) measurement reduction using Kaczmarz’s method with specified values of λ and numbers of iterations, (**j**–**l**) measurement reduction using gradient descent with specified values of λ and numbers of iterations. Zoomed-in insets show areas discussed in the text where the changes due to the choice of parameters are the most prominent.

The iterative approach described in Section 2.4.2 was applied to the sinograms of a phantom structure (Figure 9) and to sinograms of teeth (Figure 10 and Figure 11) as well. Processing results are shown for the version of the algorithm using Kaczmarz’s method for solving linear equation systems and for the version of the algorithm using gradient descent. For comparison, results of SART and positive parts of the results of filtered backprojection are shown as well. For each sinogram, the first size number is the number of sensors, while the second size number is the number of values of rotation angles.

From comparing the results, one can see that the version of the measurement reduction algorithm using Kaczmarz’s method provides better sharpness and fewer artifacts for suitable choice of parameters, but it is more sensitive to the choice of the step size parameter λ. Lower values of λ when using Kaczmarz’s method provide better results (cf. Figure 10e,g,i, as well as Figure 11e,g,i), but increase the number of iterations required for the same sharpness (cf. Figure 9e,g,i). Increasing the number of iterations for Kaczmarz’s method improves sharpness, but sometimes decreases contrast. Measurement reduction using gradient descent method shows artifacts where the object touches the inscribed circle that is the region Ω (see Figure 10j–l, right side). As mentioned before, measurement reduction using the gradient descent method leaves slight blurring compared to measurement reduction using Kaczmarz’s method. For the version of the algorithm with Kaczmarz’s method, the used values of λ are chosen as half of the value at which the numerical instability becomes prominent (∼1) or lower. For the version of the algorithm with gradient descent, the chosen values of λ are half of its maximal value and slightly below the maximum value $2/{∥A∥}^{2}$. The numbers of iterations are the numbers of sensors in the array (at least this many iterations are needed to use all data in the sinogram) and twice the number of sensors. The same circular and linear artifacts as in Figure 7 appear, although with less prominence, in Figure 10 and especially Figure 11.

In the case of the sinogram of a tooth with a lead filling (Figure 11), an artifact in the form of a bright strip at the border of the filling is visible in all processing results. This is an example of the situation in which previously mentioned additional information about material homogeneity could be useful.

Compared with SART, the measurement reduction result obtained using Kaczmarz’s method allows better discernment of details far from the image center (for example, the object’s structure at the right side of the image in Figure 10 can be seen easier).

## 4. Conclusions

Methods of the theory of computer-aided measuring systems and corresponding algorithms for improving quality of measurement reduction of computed tomography images by taking into account non-negativity of pixel brightnesses are proposed.

One method is based on combining an assumption-free image of the object and a dummy estimate. This is followed by projection onto the set of plausible images. The method is developed for the case when this set is the non-negative cone. The projection in the proposed algorithms can be easily replaced by a more complex projection routine corresponding to richer information about the object. An example of such information is discreteness of brightness values. These steps are implemented iteratively.

The other approach consists of two stages. In the first stage, a rough image estimate is obtained with methods such as backprojection. This estimate does not take into account any additional prior information. Next, at the second stage, the point spread function of blurring of the image obtained at the first stage is estimated, and the resolution is improved by local reduction taking into account the non-negative brightness of this image, similar to the first method.

The proposed methods allow processing large-dimensional data on computers with limited computing power and memory size. They do not require inversion of large matrixes except for the covariance matrix of measurement errors (in the first method), which is often diagonal. Their efficiency is demonstrated using examples of images obtained by computed tomography methods. Finally, we note that the choice of parameters of the proposed methods and their accuracy depend on the measurement model error. While it was not included in the measurement model (4), results for teeth sinograms show that its influence is comparable with the influence of the measurement noise.

## Figures and Tables

**Table 1:** Number of operations and memory consumption of sinogram processing methods.

Method	Arithmetic Operations	Consumed Memory
Algorithm 1 (local)	$\frac{2^{7}}{3}M^{6}+12J^{2}M^{2}+\frac{1}{2}J^{4}$ + UFBP	$4M^{2}+J^{2}$ + UFBP
Algorithm 2 (iterative, using Kaczmarz’s method)	$4J^{2}N_{iter}$	J(K+J)
Algorithm 3 (iterative, using Kaczmarz’s method; MR-K)	$5J^{3}N_{iter}+K$	J(2J+K)
Algorithm 2 with Landweber method (MR-GD)	$8J^{2}KN_{iter}$	Kmax{K,J}
SART (for comparison)	$3J^{3}N_{iter}+K$	J(2J+K)

Only terms with the largest powers of the number of projection angles *K*, the number of sensors *J* and the number of iterations $N_{\mathrm{iter}}$ are shown. “+ UFBP” denotes that while unfiltered backprojection is to be done before applying the algorithm, the operations and memory used in calculation of the result of unfiltered backprojection were not included in the above table. We do not consider the use of Strassen’s algorithm for matrix multiplication. Please note that the $N_{\mathrm{iter}}$ for different methods generally differ: for Algorithm 2 with Kaczmarz’s method $N_{\mathrm{iter}}∼KJ$, while for other shown algorithms $N_{\mathrm{iter}}∼K$.

## Data Availability

Data was obtained from the Laboratory for Reflectometry and Small-Angle Scattering of A. V. Shubnikov Institute of Crystallography, RAS, and are available from the authors with the permission of the Laboratory for Reflectometry and Small-Angle Scattering of A. V. Shubnikov Institute of Crystallography, RAS.

## Abbreviations

The following abbreviations are used in this manuscript:

ART	Algebraic reconstruction technique
CT	Computer tomography
FBP	filtered backprojection
MR-GD	measurement reduction using gradient descent
MR-K	measurement reduction using Kaczmarz’s method
RAS	Russian Academy of Sciences
SART	Simultaneous algebraic reconstruction
UFBP	unfiltered backprojection
w. r. t.	with respect to
